# 

**Published:** 1879-01

**Authors:** 


### Table of Contents.

### ORIGINAL COMMUNICATIONS.

Medical Societies in the Country. *By E. A. Cobleigh, M. D., Athens, Tenn.* ..... 577
An Anomaly in Obstetrics. *By A. H. Wilson, M. D., Jonesboro, Tennessee* ..... 584
Tabes Mesenterica. *By S. W. Johnson, M. D., Atlanta, Ga.* ..... 589

### REPORTS OF SOCIETIES.

Atlanta Academy of Medicine ..... 593
Pathological Society of Philadelphia ..... 594
Philadelphia County Medical Society ..... 597
Quarterly Meeting of the Rhode Island Medical Society ..... 604
Proceedings of the Essex North District Medical Society ..... 605

### SELECTIONS.

Is the Hypodermic Injection of Piles Dangerous? ..... 607
Note of some Points in Regard to the Actions and Uses of Pilocarpin and its Salts ..... 609
Case of Extra Uterine Pregnancy—Death ..... 611
The Physiology of Sleep ..... 618

### EDITORIAL.

Yellow Fever Board of Experts ..... 624
Opium Habit ..... 626

### BIBLIOGRAPHICAL.

Differential Diagnosis ..... 628
Conspectus of Organic Materia Medica and Pharmacal Botany ..... 628
Notes on the Treatment of Skin Diseases ..... 629

### EXCERPTA.

Placenta Prævia ..... 629
Prize of One Hundred Pounds for an Essay on Hydrophobia ..... 631
Dr. Walker's Modification of Sayre's Apparatus ..... 632
Why the Philadelphia County Medical Society is a Secret Society ..... 634
Innervation of the Uterus and of its Vessels ..... 634
Effects of Rhubarb and Santonin on the Urine ..... 635
Effect of Bicarbonate of Potash on the Acidity of Urine ..... 636
The Form and Contagiousness of Yellow Fever ..... 637
Ulceration of Frænum Linguæ in Pertussis ..... 638
New Method of Covering the Taste of Cod Liver Oil ..... 638
Does Quinine Affect the Hearing? ..... 638
Turpentine Vapor in Accidents from Chloroform Vapor ..... 639
Chloral as a Counter-Irritant ..... 639
Hydrophobia Cured by Oxygen ..... 639
How to Make a Poultice ..... 640
A Perfumed Solution of Iodoform ..... 640
A Remarkable Case ..... 640

